# Analyzing the Effectiveness of Finance in Supply Chain in Solving the Financing Difficulties of SMEs Based on Grey Theory Model

**DOI:** 10.1155/2022/7608937

**Published:** 2022-07-14

**Authors:** Guofeng Piao, Biyan Xiao

**Affiliations:** ^1^School of Economics and Management, Yan'an University, Yan'an 716000, China; ^2^School of Economics and Management, Weifang University of Science and Technology, Weifang 262700, China

## Abstract

Small and medium-sized enterprises (SMEs) play an indispensable role in China's national economic system, and they can play a critical role in promoting economic growth and full employment. The main reason for the disruption and impediment to the development of SME clusters is that the enterprises in the clusters are experiencing a “capital shortage,” as a result of the double contradiction between the clusters' financial benefits and the difficulties in financing. Grey theory is a relatively new concept in the field of information processing. It proposes theories and methods for processing and analyzing incomplete information systems using mathematical methods. This study uses finance in supply chain as a new research perspective to investigate the effectiveness of finance in supply chain in resolving SMEs' financing problems using the Grey theory model, with the goal of resolving SMEs' financing problems. When comparing the results of the Grey theory model and the regression analysis model, the relative error of the Grey theory model is on average 12.2% lower than that of the regression analysis model, indicating that the Grey theory model greatly improves accuracy. As a result, using the Grey theory model to solve supply chain financing and SMEs' difficulties can share risks, share credit, reduce transaction costs, weaken information asymmetry, and achieve mutual benefits and a win-win situation. It is expected to promote the development of the finance in supply chain model, mobilise enterprise enthusiasm for using finance in supply chain, improve finance in supply chain operational efficiency, and promote finance in supply chain development.

## 1. Introduction

At present, financing for SMEs is one of the major concerns of the whole society, and “supply chain financing” is a hot topic in the banking industry [1]. At present, with the improvement of capital market access standards, it is difficult for many enterprises to raise funds by issuing stocks or bonds, and in this case, the main way for enterprises to obtain sufficient funds is from banks [2]. Although the state has introduced support and incentive policies for SMEs, the effective implementation of the policies has a certain time lag effect and the effects that take time to appear [3]. In the development of SMEs, there are many problems, and the most important of which is the financing problem [4]. Domestic and foreign industrial competition is becoming increasingly fierce, and the development of SMEs is increasingly constrained by factors such as resource scarcity, energy shortage, and environmental damage [5]. The deep-rooted contradictions in the development of SMEs are becoming increasingly prominent, and the survival of SMEs is facing enormous pressure and challenges [6].

Finance in the supply chain is an important tool for SMEs to overcome their financial difficulties [7]. Corporate mergers and acquisitions are increasingly being used by companies to expand their business scale and change their development direction because of their multifaceted impact [8]. The practice and development of domestic commercial banks' supply chain finance have provided a good solution to the SMEs' financing problem [9]. This business model has broken the constraints of traditional financing and greatly expanded the credit market for SMEs, which has been recognised and imitated by an increasing number of commercial banks [10]. Although the state has eased SMEs' access to the market, it has not provided more preferential policies to help them grow [11]. In this case, establishing a special mechanism to create a new financing model to meet the financial needs of SMEs through collaboration among multiple participants or stakeholders in the credit market would be a more effective approach [12].

Grey theory [13] uses the similarity of the various factors affecting the system to determine the degree of relevance of various factors. The development of the system can be quantified by analyzing and comparing various factors to determine the most important factors in the system's and things' development [14]. Small businesses are constrained by their own capital in the continuous production process, making it difficult to organise the optimal production scale in accordance with market demand and maximise profits [15]. As a result, the preliminary work of planning the company's M&A financing, the basis of financing decision, will directly affect the ultimate success or failure of the company's M&A. Correct judgement of the macrorisks of financing and comprehensive risk analysis of the available financing methods is the preliminary work of planning the company's M&A financing, which will directly affect the ultimate success or failure of the company's M&A. According to Grey theory, the prediction of a system with both known and unknown or uncertain information is the prediction of a time-related Grey process changing in a particular direction.

The innovative points of this study are as follows:

1. It is of great practical significance to assess whether finance in supply chain can promote the development of SME clusters. The study provides a theoretical basis for it by establishing a mathematical model.
2. Focusing on the unique advantages of finance in supply chain financing model to solve the financing difficulties of SMEs, it directly reflects the specific effectiveness of finance in supply chain in the actual operation process and the inherent advantages of risk control.
3. Combining the characteristics and coupling relationship between finance in supply chain and SME clusters, a new “1 + N” pooled invoice financing method of finance in supply chain is proposed. It is innovative to connect SME cluster financing with finance in supply chain to improve financing efficiency.

This study's research framework is divided into five sections, which are organised as follows: the first section of this study introduces the research background and significance before moving on to the main work. The second part introduces the work done in supply chain finance to solve SMEs' financing issues, as well as the Grey theory model. The method of establishing the Grey theory model and the development model of finance in supply chain is sorted out in the third section, so that readers of this study can have a better understanding of the concept of analyzing finance in supply chain based on the Grey theory model to solve the financing of SMEs. The thesis' core section describes the use of the Grey theory model to analyze small business financing problems from two perspectives: serial Grey correlation analysis and sliding Grey correlation analysis. The thesis concludes with a summary of the entire work.

## 2. Related Work

### 2.1. Financial Supply Chain Financing for SMEs

With the deepening of reform and opening up, China's economy has developed rapidly and SMEs have also started to develop rapidly, becoming an indispensable and important part of China's economic system. Despite their increasingly prominent role in promoting the stable development of the national economy, fostering scientific and technological innovation, and providing a platform for employment, the number of SMEs is also increasing. However, their growth environment is not ideal, and the problem of having financial difficulties is particularly prominent. The rise of finance in supply chain has made it possible for this idea to become a reality.

Kouvelis et al. pointed out that in the UK financial system, SMEs still face financial difficulties in raising the necessary long-term capital. With the outstanding contribution of SMEs in expanding employment, solving the imbalance of regional economic development, and ensuring sustainable and stable economic growth, SMEs have become an important driving force of China's market economy [16]. According to Gaea, SME clusters create a good “green” environment for the development of finance in supply chain, and finance in supply chain provides a guarantee for the realization of the financial strategy of SME clusters [17]. Abbasi et al. proposed the net income theory, the net operating income theory, and the intermediate tradition theory as the initial theory of capital structure [18]. Luca et al. argue that various types of SMEs within an industrial cluster form an integrated supply, production, and marketing supply chain through a clear division of labor and mutual cooperation. The objective conditions are created due to the frequent capital transactions between the participants [19]. Sun et al. argued that SMEs inevitably encounter financial difficulties due to factors such as low credit levels, lack of collateral, and inadequate small and medium-sized guarantee institutions [20].

Finance in supply chain is a new financing model that has taken the world by storm in recent years. It unites core enterprises and upstream and downstream enterprises to provide flexible financing methods and effectively alleviate the financial difficulties of SMEs. Rather than assessing the production and operation status and credit risk of individual enterprises in isolation, finance in supply chain studies the financing needs of SMEs from the perspective of the whole industry chain, taking into account the core enterprises and upstream and downstream enterprises. It also considers logistics, capital flow, and information flow to achieve efficient connection.

### 2.2. Grey Theory Model

It is difficult to obtain a more complete and accurate understanding of complex systems due to the limitations of people's level of understanding, their ability to obtain information, and the existence of interactive interference between different types of information. The main characteristics of SMEs are small size, flexibility, and vitality, but they face difficulties in the development process in terms of capital, talents, and information. How to effectively extract, filter, and process information has attracted widespread attention and great importance, so Grey theory is a new discipline that has emerged.

Yao and Zhu introduced the concept of buffer operator and proposed a new method to attenuate serial shock perturbations and improve the prediction accuracy [21]. Zhou classified the expected returns of assets into two categories: risk-free returns and risky returns. The risk-free return is the risk-free rate in the capital market, and the higher the risky return is relative to the overall market risk, the higher the risk-return ratio needs to be achieved [22]. He uses function transformations to normalize the raw data and further improve the smoothness of the raw data stream. Also, theory and examples show that this mathematical transformation method is highly advantageous for improving the smoothing of the series [23]. Liu treats real security returns as the result of changes in many factors in the economy and assigns corresponding weights to each variable, assigning higher weights to factors with greater influence and less influential factors to those with greater influence [24]. Ning used the buffer operator forecasting principle to make short-term forecasts of Chinese energy consumption using a second-order weakening operator acting on the original series [25].

Under the effect of financial disincentives, there will be a “capital gap” in SME financing, resulting in an imbalance between supply and demand for capital. In the absence of supply chain support, enterprises in the group are likely to produce homogeneous and indistinguishable products, leading to fierce competition among enterprises. Based on the Grey theory model, through the coordination and integration of capital flow, logistics, and information flow, the smooth operation can be effectively ensured.

## 3. Based on the Grey Theory Model, The Idea of Finance in Supply Chain to Solve the Financing Problem of SMEs Is Analyzed

### 3.1. Establishment of Grey Theory Model

**Figure 1:**
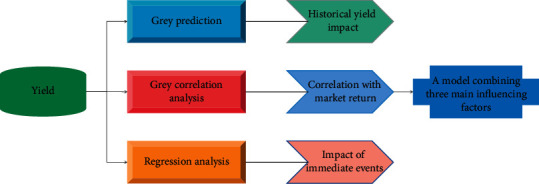
Forecast method of yield.

In cybernetics, things can usually be represented visually in different colors depending on the amount of information and whether the information is clear or not [26]. For a thing, if its information is informative and clear, then it can be said to be white [27]. If a thing has very little information and the information is vague, then it is black. What is in the middle is called Grey [28]. When considering a Grey theory model, sometimes the occurrence of certain events can lead to certain fluctuations in the returns of the event window. Therefore, the impact of events on the return must be fully considered in the Grey theory model, and the return forecasting method is shown in Figure 1.

First, to bring our Grey portfolio-based investment model closer to the real market, we refer to semiabsolute diversification risk in measuring risk. The process of model modeling involves using the original data series as solutions of the differential equation and modeling the approximate differential equation for those series that can approximately satisfy the conditions of the differential model. Assume that *S*=*S*(*i*) represents the consumer or investor surplus of a financial product and Π=*π*(*i*) represents the profit of a financial firm, both of which are functions of financial market prices. Then, the political production function is as follows:

(1) $$\begin{array}{c} M=M\left(S,\pi \right). \end{array}$$

**Figure 2:**
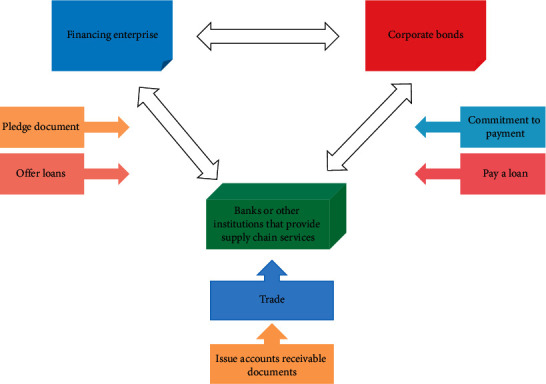
Flowchart of accounts receivable financing mode.

A necessary condition for the formation of SME clusters is the degree of association between enterprises. The characteristics, status, and role of each enterprise in the cluster, as well as the cooperative relationship between related enterprises, determine the network structure of the cluster [29]. Since many SMEs have an inadequate management system, the company's management system is not well developed and the disclosed operational information is not very complete. In this case, an information asymmetry is created [30]. Therefore, by borrowing from core enterprises and funding the actual transactions with them with supply chain financial services, we can effectively help SMEs to obtain the required loans at a lower cost and alleviate the problem of insufficient capital liquidity. In particular, when it is difficult to determine the optimal structure through visualization, comparative methods and quantitative analysis are used to determine the nuances of different structures in order to achieve the differentiation of structural strengths and weaknesses. Currently, in the actual operation of Chinese banks, together with the adoption of the receivables financing commitment method, the flowchart of the receivables financing model is shown in Figure 2.

The second is to mainly consider the case where the investor's expected return is uncertain; that is, the return on investment is represented by a Grey number. Since the expected return on assets of investors may fluctuate within a certain interval, the interval Grey number is used to represent the return on assets. In practical applications, when shock perturbations have a distorting effect on the original data stream, the buffer operator can be given priority to act on the original data stream to attenuate the perturbation distortion. The residual is the difference between the actual value and the simulated value, and the residual test actually tests the degree of deviation of the simulated value from the actual value. Let the original and simulated data be

(2) $$\begin{array}{c} x^{\left(0\right)}=\left\{x^{\left(0\right)}\left(1\right),x^{\left(0\right)}\left(2\right),x^{\left(0\right)}\left(3\right),\ldots ,x^{\left(0\right)}\left(n\right)\right\},\\ {\hat{x}}^{\left(0\right)}=\left\{{\hat{x}}^{\left(0\right)}\left(1\right),{\hat{x}}^{\left(0\right)}\left(2\right),{\hat{x}}^{\left(0\right)}\left(3\right),\ldots ,{\hat{x}}^{\left(0\right)}\left(n\right)\right\}. \end{array}$$

The residual sequence is as follows:

(3) $$\begin{array}{c} E=\left(e\left(1\right),e\left(2\right),\ldots ,e\left(n\right)\right)\\ =X\left(0\right)-\hat{X}\left(0\right). \end{array}$$

Among them

(4) $$\begin{array}{c} e\left(i\right)=x^{\left(0\right)}\left(i\right)-{\hat{x}}^{\left(0\right)}\left(i\right),\left(i=1,2,\ldots ,n\right). \end{array}$$

The cluster is a gathering place of many highly related industries, with clear division of labor and close connection, forming a “sub-network” of the supply chain. For the non-negative discrete series, the current theory describes the model by approximating the exponential form of the first-order differential equation to the exponential form of the first-order differential equation. The first stage is the primary stage, in which bank credit is introduced in the supply chain business cooperation to improve the business credit and stability of the whole supply chain. It also encourages upstream and downstream SMEs to establish long-term and stable strategic cooperation with leading enterprises to improve the operational efficiency and competitiveness of the whole supply chain. Let *X*(0)=(*x*^(0)^(1), *x*^(0)^(2),…, *x*^(0)^(*n*)) be the sequence of system behavioral characteristics, and the observed data sequence of system behavioral characteristics is as follows:

(5) $$\begin{array}{c} X\left(x\left(1\right),x\left(2\right),\ldots ,x\left(n\right)\right)=\left(x^{\left(0\right)}\left(1\right)+\varepsilon_{1},x^{\left(0\right)}\left(2\right)+\varepsilon_{2},\ldots ,x^{\left(0\right)}\left(n\right)+\varepsilon_{n}\right)=x^{\left(0\right)}+\varepsilon . \end{array}$$

Finally, the risk of the model is represented by a Grey number on top of the expected ROI, so that only one Grey number is needed for the whole model. The SMEs in the cluster have their own supply chains, distributed in various nodes. Due to the complexity of their internal relationships, the supply chains of SMEs can be single or multiple chains, cooperating and competing with each other. Grey theory defines the temporal measure of the sequence based on the open set topology and then defines the information density, the Grey derivative, and the Grey differential equation. In the finance in supply chain model, financial institutions such as banks no longer focus on individual financial companies, but on the risk of the entire supply chain and its transactions.

### 3.2. Supply Chain Financial Development Mode-“1 + *N*” Financing Service Mode

**Figure 3:**
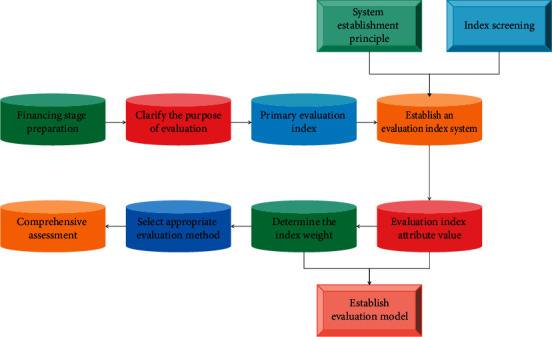
Flowchart of comprehensive evaluation of financing risk.

In the production and operation processes of SMEs, due to the time lag effect between the purchase of raw materials requiring prepayment, the sale of enterprise inventory products, and the recovery of sales payments, the enterprises have high capital flow requirements, and capital shortages occur from time to time. The development mode of finance in supply chain has innovated credit technology by providing overall service to the supply chain, integrating logistics, and information flow and closed operation, which has improved the efficiency of financial services, eased information asymmetry, and reduced transaction costs, and provided new ideas and ways to solve the financing problems of SMEs. According to the financing stage, the establishment of a more stage-specific financing risk evaluation index system provides a system guarantee for the evaluation and control of multistage financing risk, and the flowchart of comprehensive evaluation of financing risk is shown in Figure 3.

To begin with, due to a lack of short-term funds, downstream purchasers (financing companies) prefinance by confirming business on the assumption that the supply chain's presales company will buy back, relieving the pressure on downstream companies' procurement funds. Risk-averse investors are more concerned about investment risk and prefer to invest in a portfolio with a lower risk. Low risk, of course, entails a low reward. This financing model takes into account the cluster and supply chain as a whole, not only to solve individual company financing issues but also to provide financial support to individual company's upstream and downstream customers. Let the stochastic process {*X*_*n*_, *n* ∈ *T*}, whose parameter set *T* is a discrete set in time, that is, *T*={0,1,2,…}, and whose state space consisting of the entire set of possible values of the corresponding *X*_*n*_ is a discrete set of states *I*={*i*_1_, *i*_2_, *i*_3_,…}. If any integer *n* ∈ *T* and any *i*_0_, *i*_1_, *i*_2_,…, *i*_*n*+1_ ∈ *T*, then the conditional probabilities satisfy the following equation:

(6) $$\begin{array}{c} P\left\{X_{n+1}=i_{n+1}|X_{0}=i_{0},X_{1}=i_{1},\ldots ,X_{n}=i_{n}\right\}\\ =P\left\{X_{n+1}=i_{n+1}|X_{n}=i_{n}\right\}, \end{array}$$

(7) ∑j=1∞Pm,m+ni,j=1, i=1,2,…,n.

Throughout the loan financing process, the principal is the debtor of the receivables and acts as a guarantor for the upstream company, providing collateral to the bank. Once the borrower defaults on the loan, the principal firm must take over the responsibility of repaying the loan. Starting from the framework of the multivariate Markov model, it is assumed that the financial position of the firm at the time of *i*, *t* and its financial position at the time of *t*+1 satisfy the following recursive relationship:

(8) $$\begin{array}{c} x_{t+1}^{\left(i\right)}=\sum_{j=1}^{N}\lambda_{i,j}P^{\left(i,j\right)}x_{t}^{\left(j\right)},\;i=1,2,\ldots ,N. \end{array}$$

Here, *x*_*t*+1_^(*i*)^ is the probability vector of financial status.

Second, considering the conversion of existing inventory goods or operational raw materials into collateral that banks are willing to accept is a new idea for SMEs to recover bank loans. According to the evaluation criteria of indicators determined by the banking system, the evaluation indicators are graded based on the collected data and expert evaluations, so as to obtain the sample whitening number matrix for general risk assessment of supply chain financing. For the original data series with a high growth rate in the first half of the data series and small growth rate in the second half, the actual simulation error is smaller after modeling, and the construction operator attenuates or weakens the effect of the data distortion phenomenon caused by the shock perturbation shock. *X*=(*x*(1), *x*(2),…, *x*(*n*)) is the data series of system behavior characteristics, and the data series of *X* after the effect of the operator *D* is noted as follows:

(9) $$\begin{array}{c} XD=\left(x\left(1\right)d,x\left(2\right)d,\ldots ,x\left(n\right)d\right). \end{array}$$

At the same time, the financial support provided by the bank can enhance the cohesion among the companies in the cluster and make transactions more convenient. However, when choosing Greyscale generation, attention needs to be paid to choosing the appropriate Greyscale generation operation based on known data characteristics and paying attention to choosing Greyscale generation with less error.

Finally, during the sales stage, SMEs can use large parent company accounts receivables as collateral to obtain funds from banks and other financial institutions. A linear whitening weighting function is constructed based on the actual situation of supply chain financing to classify its risk into three equal low risks: low risk, average risk, and high risk. Due to the fact that the core company is a market leader with a high credit rating and good operating condition, the default risk will be reduced. The receivables generated during the subject's participation in the transaction are not invoiced, so this model is essentially indirect financing. The bank will take on the entire supply chain, including manufacturers, suppliers, and vendors, as a part of supply chain financing. This combined transaction operation eliminates many unnecessary losses and improves the fund setting. Inventory financing, also known as supply chain finance, enables SMEs to convert movable assets that banks are hesitant to accept into asset collateral via core business collateral, logistics, and storage supervision.

## 4. Application of Grey Theory Model in Analysis of Financing Problems of Small Enterprises

### 4.1. Sequence Grey Correlation Analysis

Grey theory provides a method for collecting and processing known information about systems and things in order to explore their corresponding patterns of development and change. The correlation test is a geometric test that examines the similarity between the model value curve and the actual value curve used for modeling.

**Figure 4:**
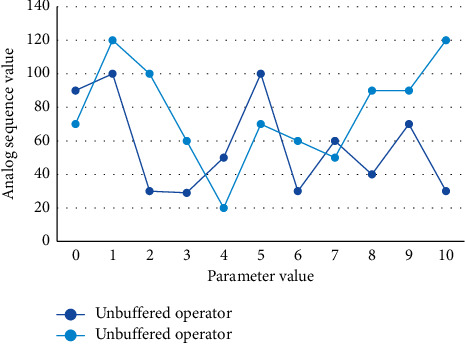
Simulation sequence diagram with or without buffer operator.

**Figure 5:**
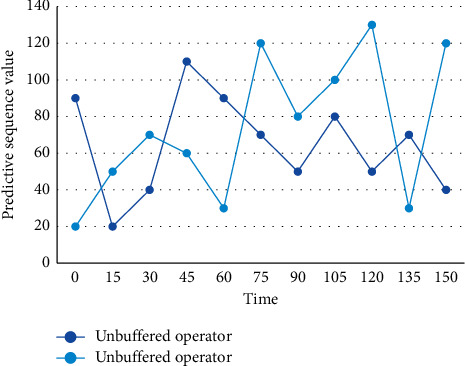
Prediction sequence diagram with or without buffer operator.

The average relative error and the predicted value are obtained by comparing the corresponding time functions after the action of the no-buffer operator and the action of the buffer operator, calculating the simulated predicted value and comparing it with the actual value, which are shown in Figures 4 and 5, respectively.

First, the method of characterizing the geometry or the dynamics of how things develop between sequences in terms of the degree of velocity difference is called absolute correlation. The degree of speed difference here is also known mathematically as the first-order slope difference. For banks, most of them choose to increase the input cost when lending to SMEs in order to reduce the risk problem, thus improving the information collection and supervision of SMEs. The predicted values of the model for 2021 after the first-order weakening of each buffer operator are listed in Table 1.

In general, the closer the geometry and the closer the trend of change, the greater the degree of correlation. The step test is a point-by-point test, the correlation test is a test of the approximation of the model to the specified function, and the posterior difference test is a test of the stochastic properties of the step distribution. The test uses the relative error size test method, which is an intuitive comparative and point-by-point arithmetic test. The predicted data are compared with the actual data to see if the relative error meets the actual requirements.

**Figure 6:**
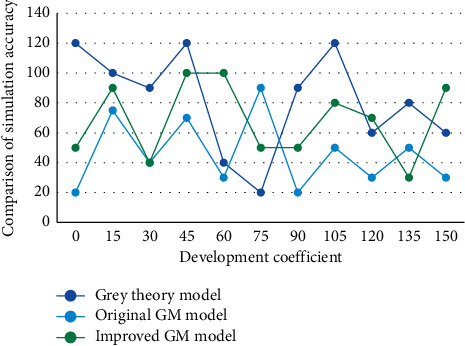
Comparison of simulation accuracy of three types of models with different development coefficients.

Second, all the Greyscale information described by the sequences can be used to highlight the “white part” and weaken the “black part” to highlight the regularity between the sequences, which establishes the optimal solution model of the whitening equation. The whitening portfolio model is a multiobjective planning problem. Therefore, the multiobjective planning model of the first whitening equation, the second whitening equation, and any whitening equation is transformed into a single-objective planning model. The supply chain financing is designed by a certain operation model so that the business income of the trust company automatically returns to a specific credit-granting bank account, which in turn repays the loan or is repaid as collateral to reduce the credit risk of the bank. A method of whitening the Grey process in the generation process has the effect of attenuating or weakening the randomness of the original data stream and plays an extremely important role in the Grey modeling process. Interval values are necessary for the theory of interval Grey number prediction, and it is necessary to provide not only the possibility of converting fuzzy numbers into interval values but also the corresponding conversion methods and algorithms. When the development coefficients take different values, the accuracy curves of the Grey theory model, the original GM model, and the improved GM model are shown in Figure 6.

Finally, the Grey series is transformed into the underlying data reflecting the essential intrinsic characteristics, that is, achieving the so-called dimensionlessness, for comparison between pairs. The risk acceptance coefficients are determined according to the different risk acceptance levels of different investors, solving the single-objective planning problem of the whitened portfolio model, and finally determining the optimal solution of the model. Cumulative generation is the accumulation of data between each series at each moment to get a new data series. Finance in supply chain integrates information, capital, logistics, and other resources, which is a scientific, personalized, and targeted financial service process. It takes the illiquid assets and the future cash flow generated by the assets in the supply chain operation link and certainty as the source of repayment and enhances the coordination of the supply chain to reduce its operation cost. From the calculation of the correlation coefficient, we can get the correlation coefficient values of the comparison series, that is, the model value series and the reference series, that is, the actual value series, which have many results at each point, and the information is too sparse to facilitate comparison. The correlation coefficient is embedded in a value, which is the sequence space before the accumulation of Greyscale correlation, called the original sequence space, and the sequence space formed after the accumulation of new data is called the generated sequence space.

### 4.2. Sliding Grey Relational Analysis

**Figure 7:**
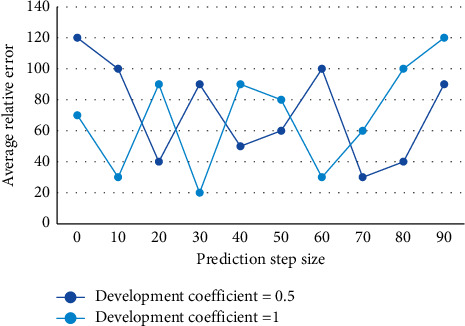
Comparison of multistep prediction accuracy of grey theory models with different development coefficients.

One of the most important indicators of national economic development is gross national product or GDP, which can reflect whether a country's economy is in the development stage or the recession stage. Due to the characteristics of Grey correlation, it makes up for the shortcomings of some mathematical and statistical methods, which are equally applicable to the sample size and whether the sample is regular or not, and the calculation is small and very convenient. At the development coefficient of 0.5 and 1, the multistep prediction accuracy of the Grey theory model is compared in the experiment, and the experimental results are shown in Figure 7.

**Figure 8:**
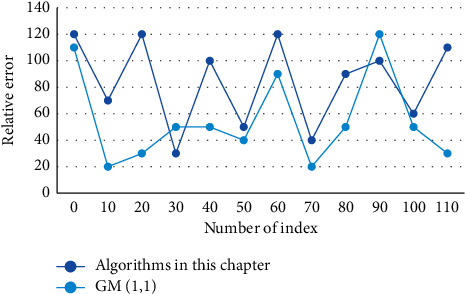
Comparison of simulation residuals between the GM (1, 1) model and the proposed model.

First, things that have some factors in common with existing things are searched for and asynchronous comparisons are made between the factors. Therefore, the concept of sliding Grey correlation is proposed to project the trend by comparing the series of different time periods (looking for analogies). In practical applications, the Grey character of portfolio investment uncertainties, the variance of risk, and expected return of portfolio investment ranges from light numbers in the classical model to interval Grey numbers are considered, as the model is closer to the real situation. The higher the smoothness of the data stream, the higher the accuracy of the established model, and vice versa, the accuracy of the model is difficult to achieve satisfactory results. Therefore, the effective evaluation coefficients of each criterion in the criteria set need to be given to increase the stability of voting and the scientificity of evaluation to obtain a more accurate ranking of the importance of members. The unbiased GM (1, 1) model eliminates the exponential bias inherent in the traditional GM (1, 1) model and the distortion of the data series when the original data series is perturbed by shocks in the traditional GM (1, 1) model. The main reason is that the unbiased model avoids jumping from the difference equation to the differential equation when modeling the GM (1, 1) model. A comparison of the simulated residuals of the GM (1, 1) model and the proposed model in this study is shown in Figure 8.

Second, the amount of the slider on the left represents the time series' advancement, while the amount of the slider on the right represents the time series' lag. Because interval Grey numbers were introduced, any set of interval Grey number whitening values in the model corresponds to a possible scenario, and the results are unreliable and could be good or bad. In general, cumulative generations can give data a degree of regularity, and if that is not enough, the number of cumulative generations can be increased. Although the requirements for meeting multiple sets of criteria are more stringent, the affiliation metric quantified values combining group consensus and individual understanding can be more accurately reflected among multiple sets of criteria, and the discriminative ability will improve. We choose the Grey theory model for forecasting and the regression analysis model for forecasting three stocks of the same type, assuming no events will affect the stock price during the forecast period. The relative errors are compared in Table 2.

Comparing the results of the Grey theory model and the regression analysis model, we can see that the relative error of the Grey theory model is on average 12.2% lower than that of the regression analysis model, which greatly improves the measurement accuracy.

Finally, overruns and lags are unique time-series properties that are only used as reference indicators during the correlation investigation process and do not represent good overruns or lags. The processed generative series are used to create a Grey generative model, which is then compared to the GM (1, 1) model created directly from the original data. The simulated prediction series can be obtained after recovering the model. To understand the decision risk, it is necessary to find the best and worst solutions for linear interval planning problems, as well as provide decision-makers with a range of effective solutions under certain conditions. Although some generative models have high prediction accuracy for Greyscale generated series, after recovering the original series, the prediction accuracy may be low, so the type of Greyscale generation used has an impact on the prediction accuracy. As a result, the dominance measure must take into account all of these factors in order to construct a reasonable set of evaluation criteria, and it is also necessary to consider the overall impact of different criteria sets on the evaluation problem and change the content of the criteria sets as needed to suit different evaluation criteria.

## 5. Conclusions

In the increasingly competitive modern market economy, the competition among enterprises is changing into the competition among supply chains. The development of finance in supply chain has brought hope to solve the financing problems of many SMEs in China. Finance in supply chain has fully integrated financial resources and realized model innovation, and the service scope has been extended to SMEs. The difficulty of SME financing is a classic issue with complex historical and objective reasons, while finance in supply chain is a newly emerged financing model in recent years, and it is the entry point of this study to crack the old problem with a new model, which is also the main purpose of this research. This study takes the financing of SME clusters as the research object and makes comprehensive use of theoretical knowledge such as the Grey theory model and finance in supply chain to conduct an in-depth study on the current situation of SME cluster financing. Using the Grey theory model to analyze the finance in supply chain has the unique advantage in solving the financial difficulties of SMEs. Introducing the Grey theory model can effectively weaken the information asymmetry between banks and SMEs and increase the possibility of banks to provide credit to SMEs. The Grey theory model can not only make a reasonable and accurate assessment of the overall risk situation of finance in supply chain but also predict the future development trend of the supply chain by comparing the risk levels of various indicators.

## Figures and Tables

**Table 1:** Predicted values of each buffer operator after first-order weakening.

Sequence	Average relative error	Estimated value (10,000 people)
*X*	2.89	122
*XD*_1_	4.82	371
*XD*_2_	6.77	658

**Table 2:** Error comparison of different methods.

Stock		Stock 1	Stock 2	Stock 3
Grey theory model	Estimated value	0.152	0.162	0.186
	Relative error	15.4%	12.9%	11.6%

Regression analysis model	Estimated value	0.231	0.243	0.267
	Relative error	1.56%	1.29%	0.98%

## Data Availability

The data used to support the findings of this study are included within the article.
